# P-646. Parainfluenza Infection in Hematopoietic Stem Cell Transplant Recipients – A Retrospective Review of IVIG Use

**DOI:** 10.1093/ofid/ofaf695.859

**Published:** 2026-01-11

**Authors:** Melissa Kerkelis, Megan Biggs, Maria A Mendoza, Raymund R Razonable, Nischal Ranganath

**Affiliations:** Mayo Clinic, Rochester, MN; The Mayo Clinic, Rochester, MN; Mayo Clinic, Rochester, MN; Mayo Clinic, Rochester, MN; Mayo Clinic, Rochester, MN

## Abstract

**Background:**

Hematopoietic stem cell transplant (HSCT) recipients are susceptible to infections with respiratory viruses resulting in higher morbidity and mortality. Parainfluenza virus (PIV) infection in the setting of leukopenia is associated with higher risk of lower respiratory tract infection and mortality. Intravenous immunoglobulin (IVIG) has been used as adjunctive treatment in viral respiratory infections, although data guiding its use is limited.

**Table 1:**
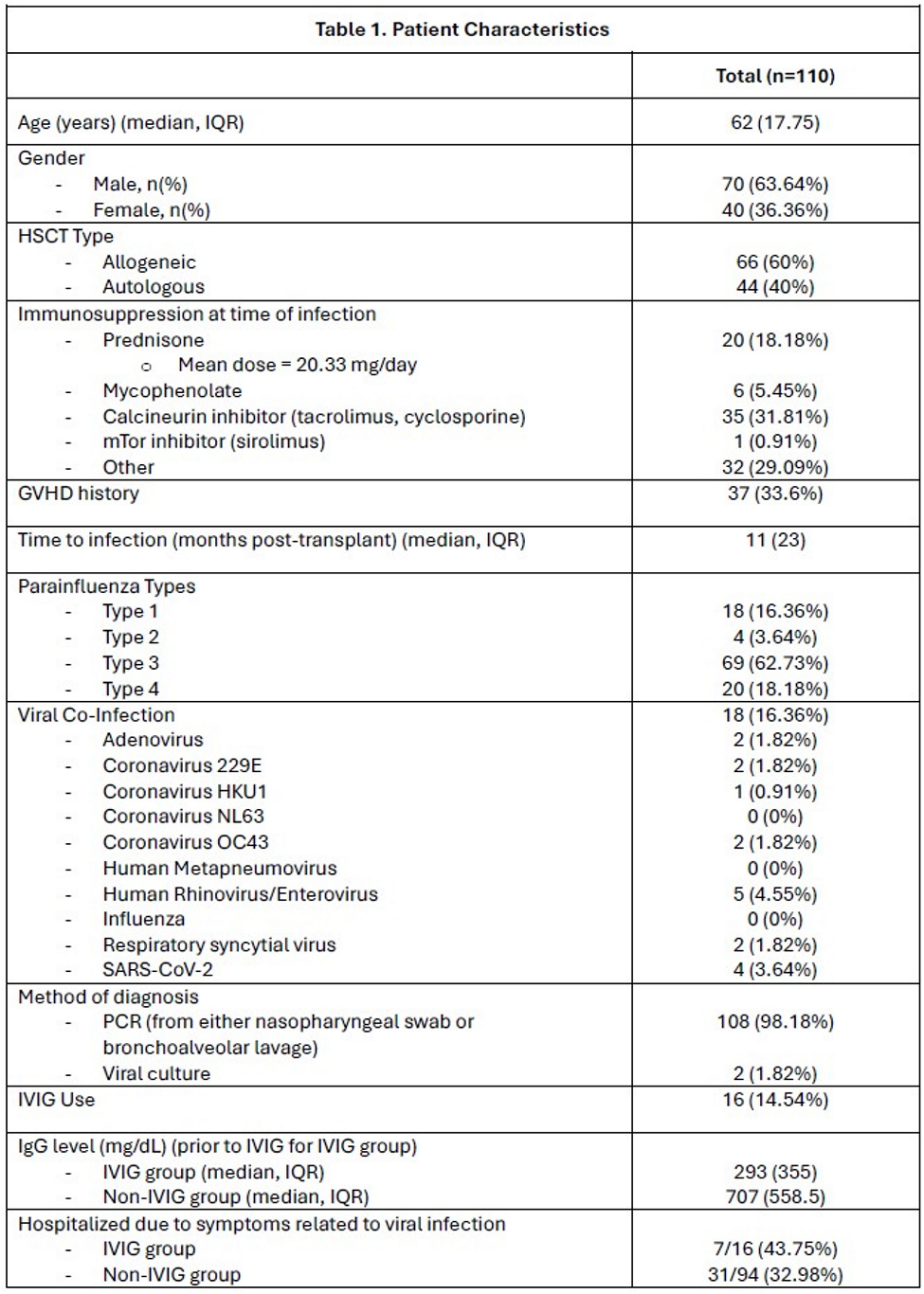
Patient Characteristics

**Table 2:**
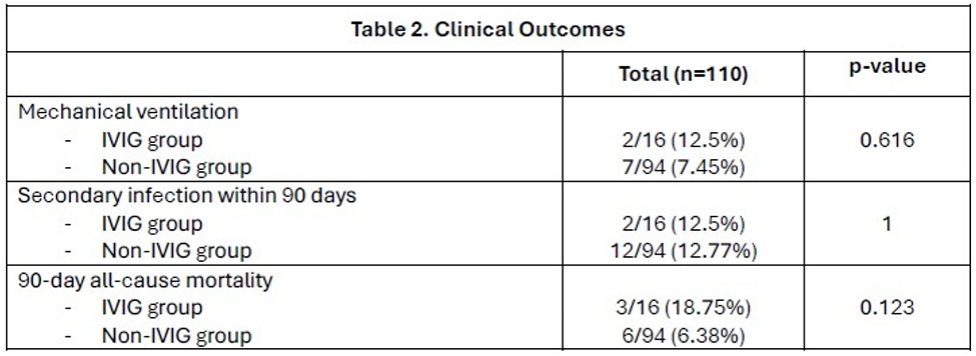
Clinical Outcomes

**Methods:**

We conducted a retrospective cohort study of adult HSCT recipients diagnosed with PIV infection at our institution from January 2014 to September 2024. Diagnosis of PIV infection was made via BioFire® Respiratory Panel multiplex polymerase chain reaction (PCR) or viral culture from nasopharyngeal or bronchoalveolar lavage. Data on clinical characteristics and outcomes were collected and reviewed.

**Table 3:**
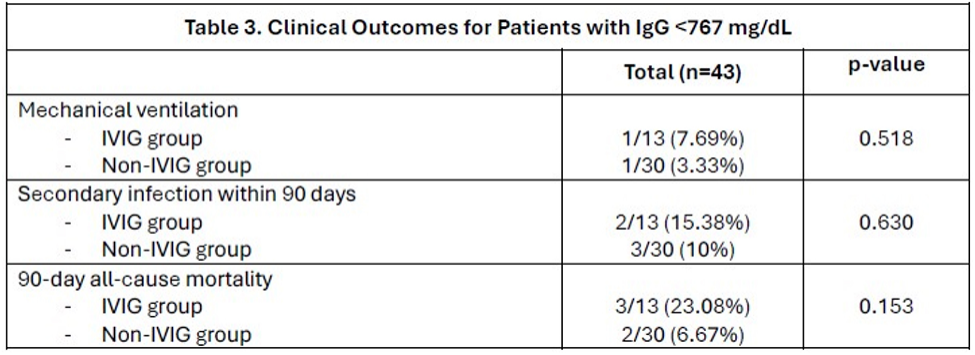
Clinical Outcomes for Patients with IgG <767 mg/dL

**Results:**

A total of 110 HSCT recipients were diagnosed with PIV infection by PCR or viral culture within the study period. Patient characteristics and clinical outcomes are summarized in Tables 1 and 2. PIV type 3 was the most common, followed by PIV type 4 and type 1. Eighteen patients (16.36%) had co-infections with another virus. A total of 16 patients received IVIG at time of PIV infection. The mean cumulative IVIG dose was 28.75 grams. Patients who received IVIG were more likely to have been hospitalized due to symptoms related to the viral infection (p=0.03) and had a lower immunoglobulin G level (p< 0.001). Overall, there was no significant difference in rates of mechanical ventilation (12.5% vs 7.45%, p=0.616), development of secondary fungal or bacterial infection (12.5% vs 12.7%, p=1), or 90-day mortality (18.75% vs 6.38%, p=0.123) among patients treated with or without IVIG, respectively. Similarly, in a subset analysis of patients with IgG level < 767 mg/dL, there were no significant difference in outcomes for patients treated with or without IVIG (Table 3).

**Conclusion:**

There were no significant differences in clinical outcomes, including rates of mechanical ventilation, secondary infections, or 90-day mortality, between HSCT patients who received IVIG and those who did not for PIV infection. Larger studies are needed to gain insight into efficacy of IVIG as adjunctive treatment in PIV infections in this population.

**Disclosures:**

All Authors: No reported disclosures

